# Peptidome Analysis of Cerebrospinal Fluid by LC-MALDI MS

**DOI:** 10.1371/journal.pone.0042555

**Published:** 2012-08-06

**Authors:** Mikko Hölttä, Henrik Zetterberg, Ekaterina Mirgorodskaya, Niklas Mattsson, Kaj Blennow, Johan Gobom

**Affiliations:** 1 Clinical Neurochemistry Laboratory, Institute of Neuroscience and Physiology, Department of Psychiatry and Neurochemistry, The Sahlgrenska Academy at University of Gothenburg, Gothenburg, Sweden; 2 Department of Occupational and Environmental Medicine, The Sahlgrenska Academy at University of Gothenburg, Gothenburg, Sweden; Moffitt Cancer Center, United States of America

## Abstract

We report on the analysis of endogenous peptides in cerebrospinal fluid (CSF) by mass spectrometry. A method was developed for preparation of peptide extracts from CSF. Analysis of the extracts by offline LC-MALDI MS resulted in the detection of 3,000–4,000 peptide-like features. Out of these, 730 peptides were identified by MS/MS. The majority of these peptides have not been previously reported in CSF. The identified peptides were found to originate from 104 proteins, of which several have been reported to be involved in different disorders of the central nervous system. These results support the notion that CSF peptidomics may be viable complement to proteomics in the search of biomarkers of CNS disorders.

## Introduction

For diseases of the central nervous system (CNS), cerebrospinal fluid (CSF) is a valuable source of biomarkers that can aid in diagnosis, provide clues to disease mechanisms and facilitate development of novel therapies. Generated through filtration of blood in the choroid plexus and by diffusion from the extracellular matrix of the brain into the ventricles [1], the CSF surrounds the brain and the spinal cord. Being in direct contact with the brain, many brain proteins diffuse into the CSF; approximately 20% of the proteins in CSF are estimated to be brain-derived [2]. CSF is produced at a rate of 500 ml/day and turns over approximately 4 times per day by drainage into the blood [3]. Thus, many ongoing processes in the CNS are reflected in the molecular composition of the CSF. Several CSF biomarkers have already been identified for various diseases and are used in research, clinical trials and clinical practice, including CSF-specific IgG immunoglobulins in multiple sclerosis, the 14-3-3 protein in Creutzfeld-Jakob's disease (CJD), the tau protein, and the β-amyloid peptides in Alzheimer's disease (AD).

While several proteomic studies have expanded our knowledge of the CSF protein composition [1], [4]–[14], there are comparatively few reports on the CSF peptidome. A few recent studies have identified a large number of endogenous peptides in the CSF [14]–[17]. Several truncated forms of these peptides and the proteins they derive from are involved in diverse biological processes, e.g., degeneration/regeneration, neuronal damage, growth, development, and learning [2]. Others may reflect the proteolytic activity in the CNS that leads to their formation through metabolic processing [16]. Studies have shown that peptide patterns can discriminate between different forms of cancer in serum [18]–[20] and discriminate AD patients from controls in both serum and CSF [21], [22].

Compared to CSF proteomics, the pursuit of the CSF peptidome is also motivated for analytical reasons. The CSF protein composition spans a concentration range of over ten orders of magnitude and is dominated by a small number of highly abundant proteins, most notably albumin, which accounts for over 60% of the total protein content [23]–[25]. Compared to albumin, for example, the concentration of the tau protein, a marker of neuronal degradation, is a million-fold lower. Global proteomic workflows generally have a strong bias towards detecting proteins of high abundance. The reasons for this are both the dynamic range of the mass spectrometric instrumentation, as well as the limited loading capacity of the separation techniques used upstream of the mass spectrometer. This requires the use of extensive sample prefractionation and sample enrichment for the detection and identification of low abundant proteins. The complexity of such workflows results in long analysis times and often compromises the analytical reproducibility, thereby hampering their use in clinical proteomic studies, in which comparative analysis of large sample sets are required. The peptides in CSF, in contrast, can be isolated relatively easily. Yuan et al demonstrated that ultrafiltration is an effective method for isolating the low molecular weight fraction (<5 kDa) of the human lumbar CSF proteome [17]. Using this strategy, Zougman et al analyzed the CSF peptidome and proteome in depth, and found 563 endogenous peptides originating from 91 proteins [14].

Here we employed nano-LC coupled to off-line to matrix-assisted laser desorption/ionization (MALDI) MS for analyzing CSF peptides in the mass range 700–5,000 Da. A method based on ultrafiltration for preparation of peptide extracts from CSF was optimized. This workflow is aimed to be suitable for comparative analysis of large clinical sample sets, necessitating a fast and simple sample preparation.

## Results and Discussion

### Sample preparation

The CSF peptidome constitutes only a minor fraction of the total protein contents of CSF. Several known bioactive peptides are present at concentrations in the pg/ml range, requiring the analysis of a few hundred microlitres of CSF to detect them by mass spectrometry. Because of the limited loading capacity of nano-LC columns (<1 µg), it is necessary to enrich the peptide fraction. Ultrafiltration using molecular weight cut-off (MWCO) filters provides a simple means to achieve this. Filters of different cut-off sizes (10 kDa, 30 kDa, 50 kDa) were evaluated. 50 kDa filters resulted in permeability of significant amounts of albumin (data not shown). While 10 kDa filters efficiently removed albumin, they retained a large part of the peptides in the mass range of interest (data not shown). According to the product documentation for the filters the recovery of a peptide of 1,350 Da is only approximately 76%. 30 kDa filters were found optimal for our application, achieving efficient removal of albumin, the most abundant CSF protein, without compromising peptide recovery.

**Figure 1:**
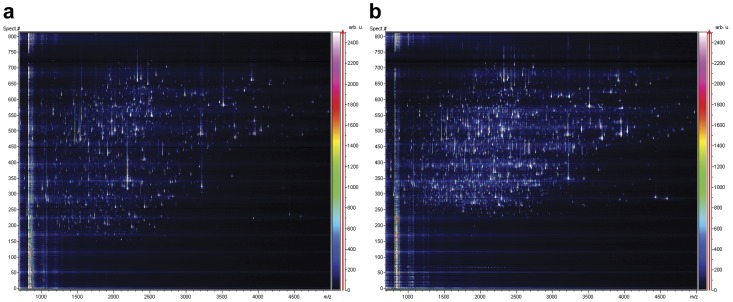
LC-MS profiles of CSF peptides. The LC retention time is shown on the y-axis and the *m/z* on the x-axis. (a) Without sample pretreatment, 2,445 compounds were detected. (b) When adding 20% ACN prior to the ultrafiltration step, the number increased to 3,543.

From studies in plasma and CSF it is well known that many peptides bind to larger proteins and may therefore be retained in the ultrafiltration step [15], [26]–[28]. To improve the recovery of such peptides we investigated the effect of pretreating CSF samples with different concentrations of acetonitrile (ACN) and formic acid (FA), in order to dissociate the peptides from the carrier proteins prior to the ultrafiltration step. A CSF pool was divided into several 500 µl aliquots to which ACN or FA were added at different concentrations as described in the method section. The filtrates were analyzed by LC-MALDI MS and evaluated on the basis of number of detected compounds. Without any sample pretreatment, 2,445 compounds were detected (Figure 1 a). In a CSF sample treated with 20% ACN, the number of detected compounds increased to 3,543 (Figure 1 b). When the concentration of ACN was increased to 40% the amount of peptides in the flow through dropped significantly. A possible explanation for the observed decrease is that at this high ACN concentration, a large part of the sample proteins precipitate, resulting in co-precipitation of peptides. The highest numbers of compounds were detected when the CSF was incubated with 20% ACN. Addition of FA up to 5% to the samples also increased the number of detected compounds, however less than ACN. Increasing the FA concentration to 10% did not yield any additional improvement. Combining ACN and FA yielded poor results.

### Peptide identification

Identification of endogenous peptides by sequence database searching is more challenging than identification of proteins in tryptic digests. Because the peptides are not produced by cleavage by one specific protease, restrictions regarding enzyme cleavage specificity cannot be applied in the database search, increasing the number of peptide sequences to consider in the search by a factor of 100–1,000, and thereby decreasing the significance of the matches in the database searches. Performing a database search with Mascot and filtering for a false discovery rate (FDR) of 1% (corresponding to a Mascot MOWSE score of 41 in the search) resulted in 596 peptide identifications. However, upon inspection of peptide hits with lower scores it was found that several of these could be confirmed based on the characteristic fragment ions arising from the presence of specific amino acids and the location of charged residues in the amino acid sequence. For singly charged peptide ions, strong charge-remote fragmentation C-terminally to aspartic acid and, to somewhat lesser degree, glutamic acid for peptides containing arginine, produces characteristic *b*- or *y*- fragment ions, depending on the location of the arginine [29], [30]. Prominent cleavage N-terminally to proline residues also produces strong *b*- or *y*- ions. Peptides that display these fragmentation characteristics often score poorly in database searches due to their selective fragmentation. However, these diagnostic fragment ions provide means to manually evaluate peptide specific matches retrieved by database searches.

For peptide identification, manual validation was performed on all database search results retrieved using a relatively low score threshold: ion score >15 and at least one peptide per protein with ion score >27. With these settings, a total of 4,185 peptide matches were retrieved. Manual validation was based primarily on the fragmentation rules discussed above for peptides containing aspartic acid/glutamic acid and arginine, and peptides containing proline. Upon inspection of the raw spectra, errors in the automatic peak detection were corrected, sometimes increasing the number of assigned peaks. In some cases validation was supported by similarity of the fragment ion pattern with that of an already identified peptide, covering partly the same amino acid sequence.

**Figure 2:**
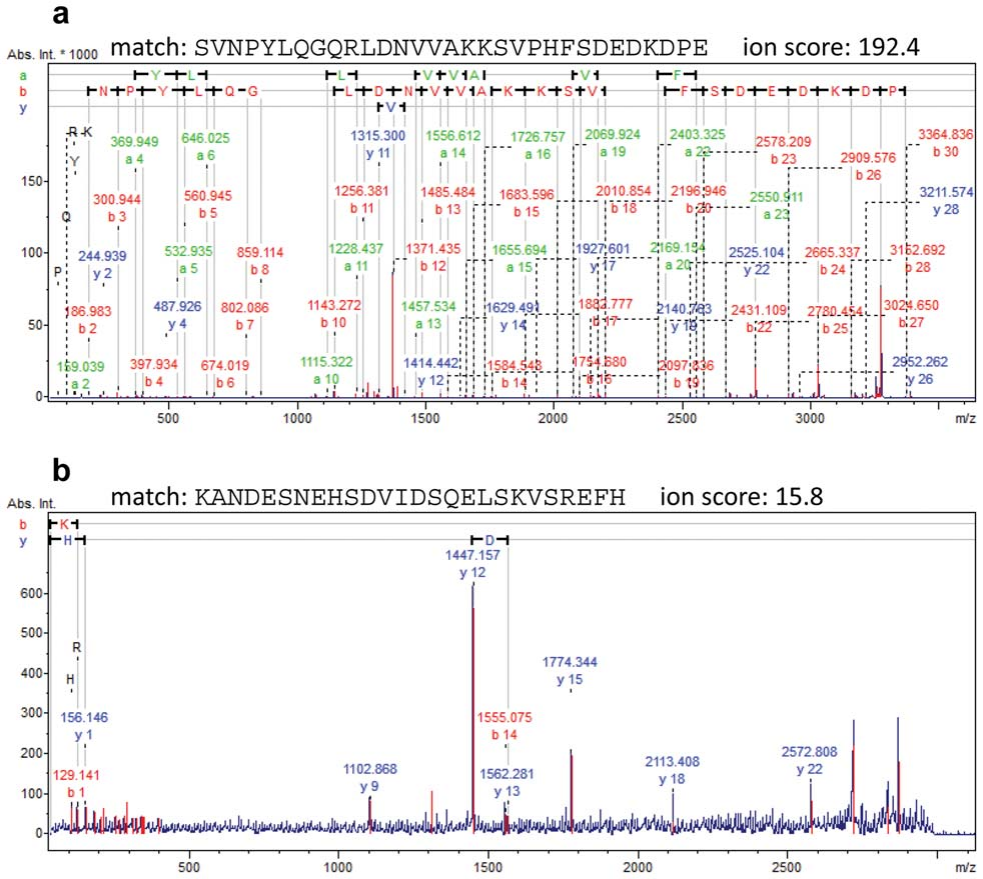
Peptide identification. (a) MS/MS spectrum of an ion of *m/z* 3511.7738 matching the peptide SVNPYLQGQRLDNVVAKKSVPHFSDEDKDPE from Neuroendrocrine protein 7B2 with an ion score of 192.4. Charge-remote fragmentation C-terminally to Asp-12, Asp-25, Asp-27, and Asp-29 due to the prescence of arginine at the N-terminal part of the peptide (Arg-10), results strong corresponding *b*-ions. (b) MS/MS spectrum of an ion of *m/z* 3000.3891 matching the peptide KANDESNEHSDVIDSQELSKVSREFH with an ion score of 15.8. Charge-remote fragmentation is observed C-terminally to Asp-14, Asp-11, Glu-8 (y-18), Glu-17 (y-9), in this case giving rise to the corresponding strong y-ions, y_12_, y_15_, y_18_, and y_9_, respectively, due to the presence of Arg-23 near the C-terminus of the peptide.

Figure 2 shows two examples illustrating the application of the described validation criteria for a strong and a weak peptide match. The fragment ion spectrum of *m/z* 3511.7738 (Figure 2 a) matched the peptide SVNPYLQGQRLDNVVAKKSVPHFSDEDKDPE from Neuroendrocrine protein 7B2 with an ion score of 194.9. Charge-remote fragmentation C-terminally to Asp-12, Asp-25, Asp-27, and Asp-29 due to the presence of arginine at the N-terminal part of the peptide (Arg-10) results in abundant corresponding *b*-ions, confirming the assignement of the peptide. The lowest scoring peptide match which was validated was the peptide KANDESNEHSDVIDSQELSKVSREFH (*m/z* 3000.3891) derived from Osteopontin, which received an ion score of 15.8. Prominent charge-remote fragmentation is observed C-terminally to Asp-14, Asp-11, Glu-8 (y-18), Glu-17 (y-9), in this case giving rise to the corresponding strong y-ions, y_12_, y_15_, y_18_, and y_9_, respectively, due to the presence of Arg-23 near the C-terminus of the peptide. Despite that the spectrum in the latter example only contains a few fragment ion signals the predictability of the fragment ion peak pattern based on the matched peptide sequence provides evidence for the correctness of the identification.

**Figure 3:**
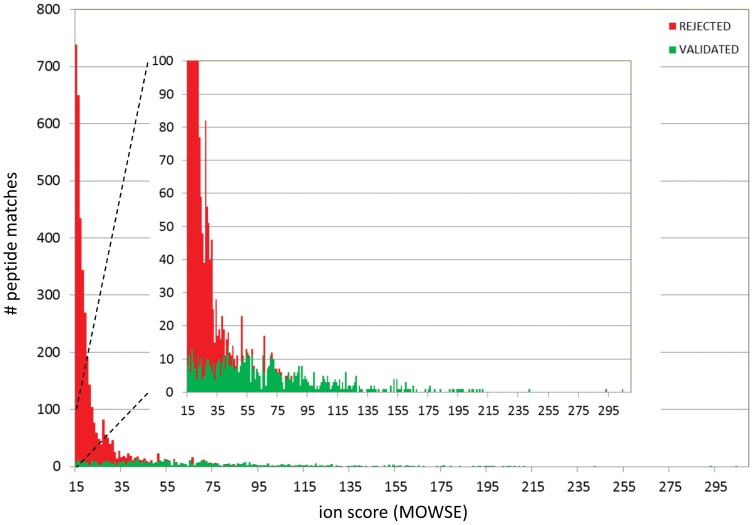
Stacked histogram of all peptide matches as a function of ion score. Peptide matches were retrieved that fulfilled the criteria: ion score >15 and at least one peptide match with ion score >27 per protein. The coloured segments indicate the number of validated (green) and rejected (red) peptide matches.

A histogram of the number of manually validated and rejected peptide matches as a function of ion score (Figure 3) shows that quite a large number of peptide hits with low ion score could be validated. A total of 730 peptide matches were validated. The 22% increase of assigned peptides by manual evaluation compared to the 596 peptides identified with an FDR of 1% in the Mascot database search is significant and suggests that, particularly for the analysis of singly-charged ions, search algorithms may be improved by implementing amino acid- and charge-based fragmentation schemes in their scoring algorithms.

The identified peptides are listed in Table S1. The identifications comprised 626 unique peptide sequences (not taking into account post-translational modifications) originating from 104 proteins (Table 1). The mass spectrometric data is available via the PRoteomics IDEntifications database [31] (PRIDE, http://www.ebi.ac.uk/pride/, Accession number 24353).

**Table 1:** List of identified proteins from the analysis of the CSF peptidome using LC-MALDI MS/MS.

Entry name	Protein name
PTPRR_HUMAN	Receptor-type tyrosine-protein phosphatase R isoform 1 precursor
NBL1_HUMAN	Neuroblastoma suppressor of tumorigenicity 1 isoform 1
PLM_HUMAN	Phospholemman precursor
CADH2_HUMAN	Cadherin-2 preproprotein
THRB_HUMAN	Prothrombin preproprotein
A4_HUMAN	Amyloid beta A4 protein isoform h precursor
ADML_HUMAN	ADM precursor
TICN3_HUMAN	Testican-3 isoform 1
VTNC_HUMAN	Vitronectin precursor
CXL16_HUMAN	C-X-C motif chemokine 16
7B2_HUMAN	Neuroendocrine protein 7B2 isoform 2
OSTP_HUMAN	Osteopontin-D
CSTN1_HUMAN	Calsyntenin-1 isoform 2
TTHY_HUMAN	Transthyretin precursor
SODC_HUMAN	Superoxide dismutase [Cu-Zn]
SORC2_HUMAN	VPS10 domain-containing receptor SorCS2 precursor
CD99_HUMAN	CD99 typeII
FIBB_HUMAN	Fibrinogen beta chain isoform 1 preproprotein
CO3_HUMAN	Complement C3 precursor
TYB10_HUMAN	Thymosin beta-10
FIBG_HUMAN	Fibrinogen gamma chain isoform gamma-A precursor
PGCB_HUMAN	Brevican core protein isoform 1
SAA3_HUMAN	Putative serum amyloid A-3 protein
APLP2_HUMAN	Amyloid-like protein 2 isoform 4 precursor
TENR_HUMAN	Isoform 2 of Tenascin-R
AACT_HUMAN	Isoform 3 of Alpha-1-antichymotrypsin
FXYD6_HUMAN	FXYD domain-containing ion transport regulator 6 precursor
SCG1_HUMAN	Secretogranin-1 precursor
COIA1_HUMAN	Isoform 3 of Collagen alpha-1(XVIII) chain
ANGL2_HUMAN	Angiopoietin-related protein 2 precursor
DKK3_HUMAN	Dickkopf-related protein 3 precursor
C99L2_HUMAN	CD99 antigen-like protein 2 isoform 3 precursor
CAD19_HUMAN	Cadherin-19 preproprotein
FBLN3_HUMAN	Isoform 4 of EGF-containing fibulin-like extracellular matrix protein 1
SE6L1_HUMAN	Seizure 6-like protein isoform 2 precursor
ANFC_HUMAN	C-type natriuretic peptide precursor
AUGN_HUMAN	Augurin precursor
NPTXR_HUMAN	Neuronal pentraxin receptor
TRH_HUMAN	Prothyroliberin precursor
PCSK1_HUMAN	ProSAAS precursor
SFRP4_HUMAN	Secreted frizzled-related protein 4 precursor
ITIH5_HUMAN	LLLL311
FMOD_HUMAN	Fibromodulin precursor
MIME_HUMAN	Mimecan preproprotein
SPRL1_HUMAN	SPARC-like protein 1 precursor
ECM1_HUMAN	Extracellular matrix protein 1 isoform 3 precursor
CART_HUMAN	Cocaine- and amphetamine-regulated transcript protein
APOE_HUMAN	Apolipoprotein E precursor
NSG1_HUMAN	Neuron-specific protein family member 1
PRIO_HUMAN	Isoform 2 of Major prion protein
GELS_HUMAN	Gelsolin isoform a precursor
PTGDS_HUMAN	Prostaglandin-H2 D-isomerase
PTPRZ_HUMAN	Isoform Short of Receptor-type tyrosine-protein phosphatase zeta
A2AP_HUMAN	Alpha-2-antiplasmin isoform a precursor
P3IP1_HUMAN	HGFL(S) protein
CADM3_HUMAN	Isoform 3 of Cell adhesion molecule 3
SCG2_HUMAN	Secretogranin-2 precursor
IBP5_HUMAN	Insulin-like growth factor-binding protein 5 precursor
A1ATR_HUMAN	Putative alpha-1-antitrypsin-related protein
GP158_HUMAN	Probable G-protein coupled receptor 158 precursor
SCG3_HUMAN	Secretogranin-3 isoform 1 precursor
FIBA_HUMAN	Fibrinogen alpha chain isoform alpha preproprotein
GPR37_HUMAN	Probable G-protein coupled receptor 37 precursor
CSF1_HUMAN	Macrophage colony-stimulating factor 1
TICN1_HUMAN	Testican-1 precursor
SFRP3_HUMAN	Secreted frizzled-related protein 3 precursor
CMGA_HUMAN	Chromogranin-A preproprotein
NCKX2_HUMAN	Sodium/potassium/calcium exchanger 2 isoform 2
ITA7_HUMAN	Isoform Alpha-7X2DB of Integrin alpha-7
RNAS1_HUMAN	Ribonuclease pancreatic precursor
A1AT_HUMAN	Isoform 3 of Alpha-1-antitrypsin
PENK_HUMAN	Proenkephalin-A preproprotein
SMS_HUMAN	Somatostatin preproprotein
CO4A_HUMAN	Complement C4-A
IGF2_HUMAN	Isoform 2 of Insulin-like growth factor II
APLP1_HUMAN	Amyloid-like protein 1 isoform 1 precursor
PTPR2_HUMAN	Isoform 3 of Receptor-type tyrosine-protein phosphatase N2
TKNK_HUMAN	Tachykinin-3 isoform 2 preproprotein
MOG_HUMAN	Isoform 9 of Myelin-oligodendrocyte glycoprotein
CYTC_HUMAN	Cystatin-C precursor
CLUS_HUMAN	Isoform 5 of Clusterin
PCDG3_HUMAN	Protocadherin gamma-A3 isoform 1 precursor
XYLT1_HUMAN	Xylosyltransferase 1
ETBR2_HUMAN	Endothelin B receptor-like protein 2 precursor
CSF1R_HUMAN	Macrophage colony-stimulating factor 1 receptor precursor
PNOC_HUMAN	Prepronociceptin preproprotein
SPRC_HUMAN	SPARC precursor
SORT_HUMAN	Sortilin isoform 1 preproprotein
ITM2B_HUMAN	Integral membrane protein 2B
TYB4_HUMAN	Thymosin beta-4
NCAM1_HUMAN	Neural cell adhesion molecule 1 isoform 4 precursor
SYT11_HUMAN	Synaptotagmin-11
SELPL_HUMAN	P-selectin glycoprotein ligand 1 isoform 2
PRR24_HUMAN	Proline-rich protein 24
VGF_HUMAN	Neurosecretory protein VGF precursor
MGP_HUMAN	Matrix Gla protein isoform 2 precursor
CSTN3_HUMAN	Calsyntenin-3 precursor
HPT_HUMAN	Haptoglobin isoform 1 preproprotein
HEG1_HUMAN	Isoform 2 of Protein HEG homolog 1
CAD11_HUMAN	Isoform 2 of Cadherin-11
CCKN_HUMAN	Cholecystokinin preproprotein
B2MG_HUMAN	Beta-2-microglobulin precursor
NPY_HUMAN	Pro-neuropeptide Y preproprotein
CGRE1_HUMAN	Cell growth regulator with EF hand domain protein 1
ALBU_HUMAN	Serum albumin preproprotein

### Comparisons with other studies

**Figure 4:**
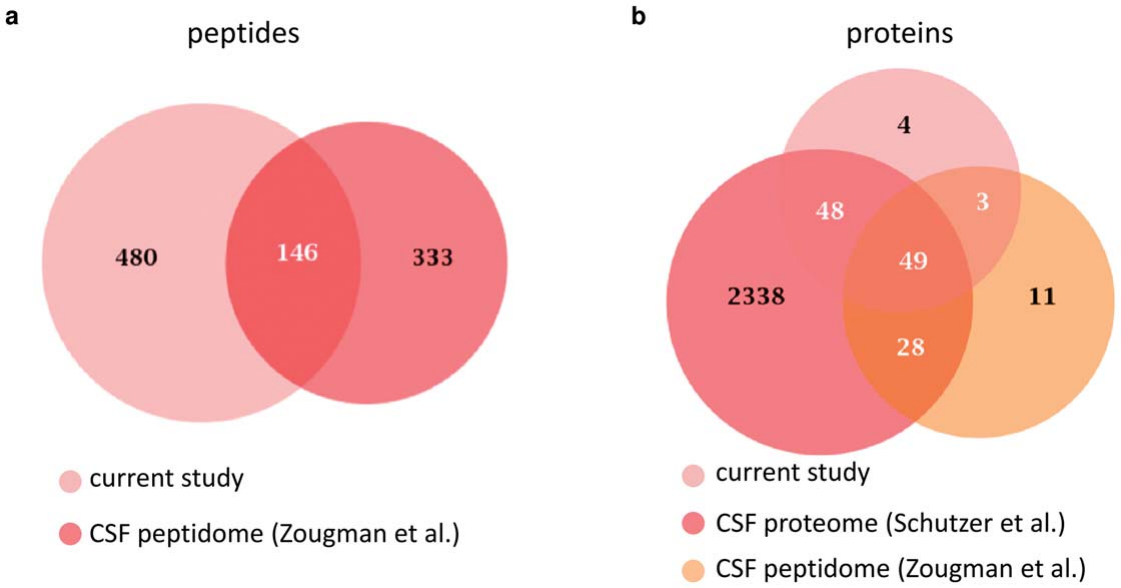
Comparison of the peptides identified in the current study to other published peptidomic and proteomic data. Only 23% of the peptides identified in the current study were part of the peptide set reported by Zougman *et al.* [14]. Comparison of the proteins represented by the endogenous peptides identified in the current study and in the study by Zougman *et al.* with the proteins identified in the CSF proteomic analysis by Schutzer *et al.* [7] (b) reveals that both peptide sets have a high degree overlap with the proteomic set.

Comparing the peptides identified in our study to those identified in the study by Zougman *et al.* [14], one of the most comprehensive CSF peptidomic studies to date, showed that only 23% of the peptides identified in our study were present in the other data set (Figure 4 a). The large difference between the data sets may be attributed to the different ionization techniques used in the two studies (MALDI vs. ESI), to differences in the method used for preparation of the peptide extracts, and that different CSF samples were analyzed.

To assess how the CSF peptidome compares to the CSF proteome, we compared the proteins represented by the endogenous peptides identified in the current study and in the study by Zougman *et al.* with the extensive CSF proteomic analysis by Schutzer *et al.* comprising 2,462 proteins [7] (Figure 4 b). Both peptidomic data sets have high overlap with the proteomic set. Thus, on a protein level, the peptidomic approaches used in the two studies mainly identifies peptides derived from the same proteins that are found by global proteomic analyses.

### Biological functions of the identified proteins

While the significance of the endogenous peptides in CSF is still largely unknown, several of the precursor proteins from which they derive are associated with various brain disorders and known cellular processes in the brain. A few examples are given below. While the presence in CSF of all of these proteins has been reported previously [7], several of the endogenous peptides derived from these proteins are reported for the first time.

#### Amyloid beta peptides

**Figure 5:**
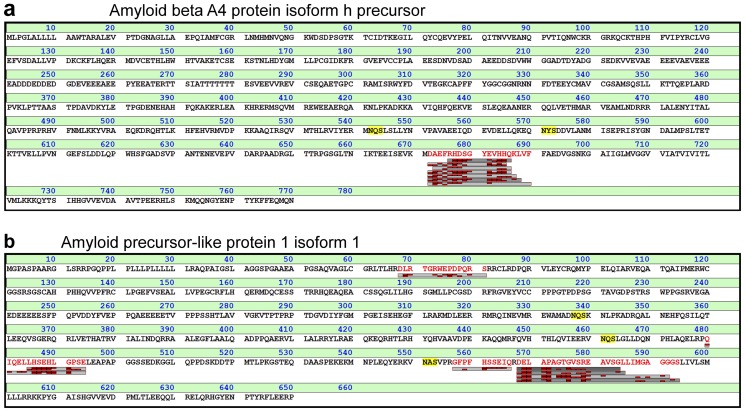
Identified endogenous peptides from (a) Amyloid beta A4 protein isoform h precursor (A4_HUMAN, commonly referred to as APP) and (b) from Amyloid-like protein 1 isoform 1 (APLP1). **T**he peptides identified from A4_HUMAN are all located within the β-amyloid peptide, starting at or in proximity of the N-terminus of the peptide, defined by the BACE cleavage site. Correspondingly, four of the peptides identified in APLP1 are generated by BACE cleavage C-terminally to Arg-567.

The amyloid beta A4 protein (amyloid precursor protein, APP) plays a central role in the pathophysiological processes in AD, which is characterized by progrediating neuronal degeneration with amyloid deposits [32]. By enzymatic processing of APP, the peptide amyloid beta (Aβ) 1–42 is generated which is highly prone to aggregation and is the major constituent of the amyloid plaques that form in the brain. Aβ1–42 is also used as a CSF biomarker for AD [33]. In this study, we detected Aβ1–14, 1–15, 1–16, 1–17, 1–19, and 5–15 (Figure 5 a). The existence of several of these fragments have been recently reported in CSF in a study using immunoprecipitation in combination with mass spectrometry [34]. The C-termini of these fragments span the α-secretase cleavage site and may thus be markers of non amyloidogenic APP processing that may be protective from AD [35]. The identification of Aβ5–15 is the first report of this truncated form in CSF. The identification of Aβ peptides starting at position 5 is especially interesting since such peptides may represent the activity of an APP processing pathway that is up-regulated after inhibition of the major Aβ producing enzyme BACE1 [36], [37] and CSF Aβ5-X peptides may be useful as pharmacodynamic markers in trials of BACE1-inhibitors [38].

#### APLP1

CSF also contains several amyloid-like proteins, which have sequence homology to APP, and that to some extent undergo similar processing. One such protein is the amyloid precursor-like protein 1 (APLP1). APLP1 undergoes processing by BACE and γ-secretase generating Aβ-like peptides of which one has been shown to have potential value as a biomarker for AD [39]. Several of these Aβ-like peptides were also identified in our study, four of which have N-termini at the BACE cleavage site (Arg-167) in APLP1 (Figure 5 b). The peptides derived from APLP1 and APP may thus be used in parallel in clinical studies to monitor the activity of these secretases to investigate if similar patterns of peptides are affected in different disease or to monitor effects of pharmaceutical compounds that target a given enzyme.

#### CART

Another interesting finding is the identification of fragment 28–36 of cocaine- and amphetamine-regulated transcript (CART), covering part of the amino acid sequence of CART(1–39). This peptide is involved in regulating many processes including body weight, reward and endocrine functions [40]. CART is together with NPY believed to regulate the leptin-mediated feeding response [41].

#### NPY

Neuropeptide Y is a highly abundant neuropeptide and a potent neuromodulator involved in several different processes, e.g. hunger, stress response, cardiovascular function, and circadian rhythms [42]. Three peptides were identified that span part of the NPY 1–36 sequence: NPY 1–20, 3–22, and 5–22, none of which have been previously reported in CSF. It is believed that the intact NPY 1–36 is the active peptide although it has not been shown in intact form in CSF.

#### MOG

From myelin oligodendrocyte glycoprotein (MOG) we identified six peptides spanning part of the extracellular region with a common cleavage site C-terminally to the amino acid at position 81. MOG is found on the surface of the myelin sheath and has an yet unclear role in multiple sclerosis where autoantibodies against the protein are found [43]. The levels of autoantibodies against MOG seem to be an indicator of disease intensity.

#### Granins

**Figure 6:**
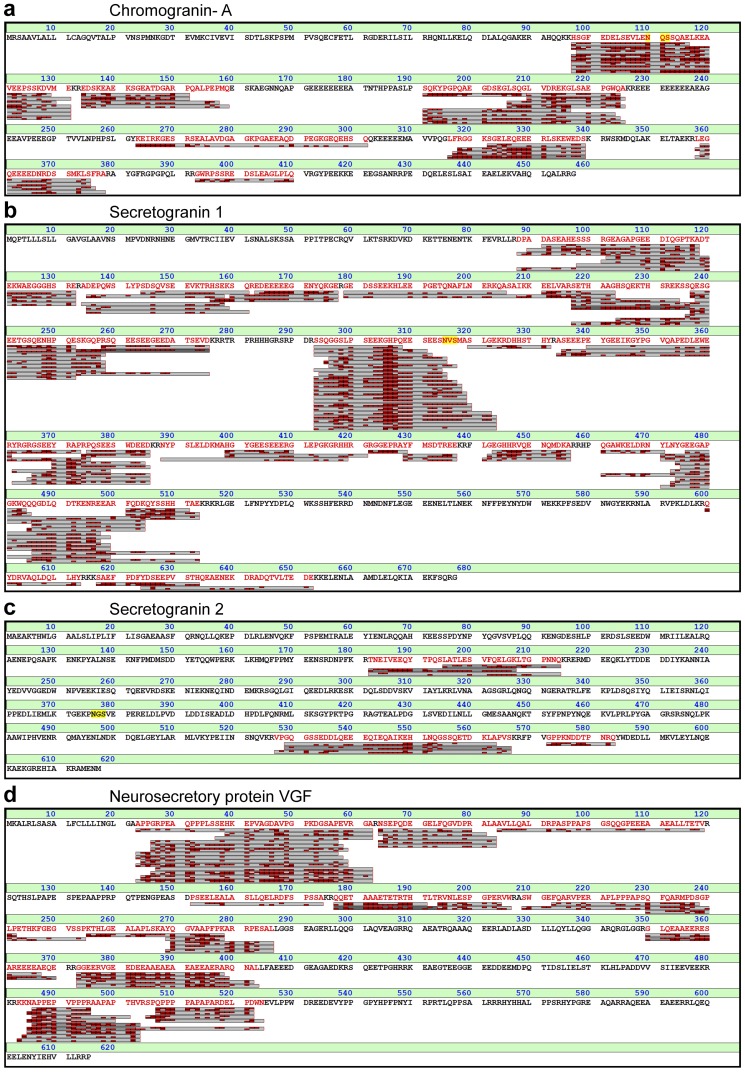
Peptides identified from the Granin family. (a) Chromogranin- A (CMGA), (b) Secretogranin 1 (SCG1), (c) Secretogranin 2 (SCG2), (d) Neurosecretory protein VGF (VGF).

Peptides from the granin family have been discussed as CSF biomarkers for various diseases such as AD, multiple sclerosis, schizophrenia, and depression [44]. The granins (Chromogranin-A (CMGA), Secretogranin-1 (SCG1), Secretogranin-2 (SCG2), Secretogranin-3, 7B2, NESP22, proSAAS, and VGF) are involved in regulated delivery of several key factors in CNS such as neurotransmitters, hormones and growth factors. Peptides from Neurosecretory protein VGF have been associated with different brain disorders, such as VGF_26–62_ [45] with decreased levels in frontotemporal dementia; VGF_378–397_ [46], [47], with decreased levels in AD; VGF_23–62_ [48], with increased levels in schizophrenia and in depression; and SCG2_529–566_ [48], with decreased levels in depression. In our study three of the peptides (VGF_26–62_, VGF_23–62_, and SCG2_529–566_) were found both as intact peptides and in truncated forms, while the third VGF peptide was only found as peptides exceeding the sequence length with 1–6 amino acids. In the case of SCG2 and CMGA, an alteration in three peptides is described as potential marker for multiple sclerosis, decreased SCG1_441–493_ [49] and SCG1_306–365_ [49] and increased CMGA_194–213_ [50], [51]. SCG1_441–493_ has also been found decreased in frontotemporal dementia [45]. Although we did not identify any of SCG1_441–493_, SCG1_306–365_, or CMGA_194–213_ as intact peptides, we identified several forms spanning parts of these sites (Figure 6).

#### Prion protein

Nine fragments of the prion protein were identified. The detected peptides are fragments created after the signal peptide has been removed from the protein with some starting at amino acid position 23 and others starting more C-terminally in the octapeptide repeat region which is associated to prion disease [52]. In CJD, which is characterized by spongiform degeneration and neuronal death leading to dementia, motor dysfunction, and eventually death, the prion protein plays a critical role. When the prion protein assumes an abnormal conformation it becomes very prone to aggregation, which starts an autocatalytic cascade that eventually produces neurotoxic species of the protein [53], [54].

### Conclusion

Our results show that LC-MALDI MS can be used for monitoring large numbers of endogenous CSF peptides in sample volumes relevant to clinical studies. Several of the identified peptides derive from proteins involved in physiological and pathological processes in the CNS. The CSF peptidome contains information about peptides spanning other parts of the proteins than are found using bottom-up proteomic workflows, and may thus be a complementary strategy for identifying biomarkers of disease. Supplementing the method described here with stable isotope labeling methods for quantification, such as the *tandem mass tag* (TMT) or *the isobaric tags for relative and absolute quantitation* (iTRAQ) approaches may be a viable strategy for identification of new biomarkers in CSN disorders.

## Methods

### Ethics statement

The study was approved by the regional ethics committee at the University of Gothenburg.

### Materials

MALDI grade α-cyano-4-hydroxycinammic acid (HCCA, part no. 201344) and mass calibrants (Peptide mass calibration standard II, part no. 222570) were purchased from Bruker Daltonics. Purified water was used for all solutions (Milli-Q Plus, Millipore), except for preparation of MALDI matrix solutions, which were prepared from bottled water (part no. 39253-1L-R) from Fluka. MWCO filters were purchased from Millipore (Amicon Ultra 0.5 mL 30 K, part no. UFC503096). FA (98%, part no. 56302-10×1ML-F) was purchased from Fluka, and trifluoro-acetic acid (reagent grade, >98%, part no. T6508) from Sigma-Aldrich.

### CSF samples

CSF samples collected by lumbar puncture from patients undergoing clinical evaluation were obtained from the Clinical Neurochemistry Laboratory, Sahlgrenska University Hospital Mölndal, Sweden. To eliminate cells and other insoluble material, the CSF samples were centrifuged at 2,000×g at 4°C for 10 min. Samples were selected from patients who did not display any clear signs of neurological disorder, with respect to cell counts, albumin CSF to serum ratio and signs of intrathecal immunoglobulin production. CSF was de-identified and pooled from 3 different patients to achieve sufficient volume. All samples were stored at −80°C pending analysis.

### Sample preparation

The CSF pool was split into aliquots of 500 µl each. To the samples were added ACN (20%–40% (v/v)) or FA (5%–10% (v/v)) after which the samples were briefly vortexed and incubated for 20 minutes at room temperature. MWCO filters of 10, 30 and 50 kDa were washed with 0.5 ml H_2_O prior to use. The CSF samples where centrifuged through the filters according to the manufacturer's recommendations. The flow-through from the filters containing the peptide fraction was recovered and lyophilized. The lyophilized samples were stored at −80°C pending analysis. To dissolve the samples, 20 µl of 0.5 M HCl, 1% TFA (v/v) was added and the samples were vortexed for 30 minutes.

### Liquid chromatography

Peptides were loaded onto a trap column (Acclaim PepMap100 C_18_, 75 µm×20 mm, LC Packings) and separated with a C_18_ column (Acclaim PepMap C_18_, 75 µm×150 mm, LC packings) on an Ultimate 3000 nano-LC system (Dionex). A sample volume of 20 µl was injected and a 150 minute gradient was used for peptide separation. The mobile phases used where A, 0.05% TFA (v/v) and B, 80% ACN (v/v), 0.05% TFA. A solenoid-valve liquid dispensing robot (Instrument2, M2 Automation) was connected to the nano-LC device and was used to dispense fraction from the nano-LC separation onto a 600 µm Anchor Chip 1536 MALDI sample plate (Bruker Daltonics). The effluent from the nano-LC was mixed with HCCA matrix solution (4 g/l, 70% ACN (v/v), 0.1% TFA (v/v)) in a T-junction proximally to the capillary end, and dispensed onto the target plate. Mass calibrants mixed with HCCA matrix solution (4 g/l in 70% ACN (v/v), 0.1%TFA (v/v)) were deposited in a pattern covering the MALDI target for near-neighbor external mass calibration.

### Mass spectrometry

The fractionated samples were analyzed with a MALDI TOF/TOF (Ultraflextreme, Bruker Daltonics, Bremen) instrument operated in the positive ion mode. The analyzed mass range was 700–5,000 Da with deflection up to *m/z* 600. For MS analysis, 2,000 single shot spectra were accumulated from 10 random positions on each sample, irradiating each position with 200 laser pulses. From the MS analysis, compounds with S/N>15, were selected for MS/MS analysis. For MS/MS analysis 2,000 single shot spectra were recorded of the precursor ions and 4,000 of the fragment ions. The analyses of the MS spectra and selection of compounds for MS/MS analyses was performed using the WARP-LC software (Bruker Daltonics), which automatically executed the MS and MS/MS analysis.

### Bioinformatics

All the acquired data were processed using the ProteinScape software (Bruker Daltonics). The MS/MS data were searched using the Mascot search engine (Matrix science) against the SwissProt sequence database with the *Homo sapiens* subset considering the following variable modifications: phosphorylation, amidation, deamidation, pyroglutamic acid, oxidation, acetylation, sulfation, and oxidized as well as reduced cysteines. The error tolerance for precursor ion masses was set to 15 ppm and for fragment ion masses, 0.6 Da. All included peptides in the search were at least 7 amino acids long. A lower peptide score threshold of 15 was used. Protein hits with at least one peptide with ion score >27 were retrieved. For these, all peptide matches were manually validated. The search results were compiled into a protein list using ProteinScape. All MS/MS spectra considered for protein identification were manually validated using the BioTools software (Bruker, Bremen, Germany) according to the following rules: a) prominent cleavage N-terminally to proline, b) b and y ion pairs, c) ion series continuity d) major peaks identified as b or y ions e) resemblance of fragment ion patterns in peptide hits that cover partially the same amino acid sequence in a protein, f) abundance of y/b ions according to position of basic amino acids in the sequence g) abundant cleavage C-terminally to aspartic acid and glutamic acid if an arginine is present in the sequence.

Comparisons of datasets, protein homology filtering and cellular localization analysis were performed using the ProteinCenter software (Thermo Fischer Scientific). In the comparisons with the HUPO datasets the proteins were clustered using Homogenous Groups at a 60% level, while the comparisons against Zougman et al [14] and Schutzer et al [7] were made with clustering for indistinguishable peptides.

## Supporting Information

Table S1 **List of identified peptides from the analysis of the CSF peptidome using LC-MALDI MS/MS.**
(XLS)
